# *Locus Coeruleus* as a vigilance centre for active inspiration and expiration in rats

**DOI:** 10.1038/s41598-018-34047-w

**Published:** 2018-10-23

**Authors:** Karolyne S. Magalhães, Pedro F. Spiller, Melina P. da Silva, Luciana B. Kuntze, Julian F. R. Paton, Benedito H. Machado, Davi J. A. Moraes

**Affiliations:** 1 School of Medicine of Ribeirão Preto, Department of Physiology, University of São Paulo, Ribeirão Preto, SP Brazil; 2 School of Physiology, Pharmacology and Neuroscience, Biomedical Sciences, University of Bristol, Bristol, UK; 3 Cardiovascular Autonomic Research Cluster, Department of Physiology, Faculty of Medical and Health Sciences, University of Auckland, Auckland, New Zealand

## Abstract

At rest, inspiration is an active process while expiration is passive. However, high chemical drive (hypercapnia or hypoxia) activates central and peripheral chemoreceptors triggering reflex increases in inspiration and active expiration. The *Locus Coeruleus* contains noradrenergic neurons (A6 neurons) that increase their firing frequency when exposed to hypercapnia and hypoxia. Using recently developed neuronal hyperpolarising technology in conscious rats, we tested the hypothesis that A6 neurons are a part of a vigilance centre for controlling breathing under high chemical drive and that this includes recruitment of active inspiration and expiration in readiness for flight or fight. Pharmacogenetic inhibition of A6 neurons was without effect on resting and on peripheral chemoreceptors-evoked inspiratory, expiratory and ventilatory responses. On the other hand, the number of sighs evoked by systemic hypoxia was reduced. In the absence of peripheral chemoreceptors, inhibition of A6 neurons during hypercapnia did not affect sighing, but reduced both the magnitude and incidence of active expiration, and the frequency and amplitude of inspiration. These changes reduced pulmonary ventilation. Our data indicated that A6 neurons exert a CO_2_-dependent modulation of expiratory drive. The data also demonstrate that A6 neurons contribute to the CO_2_-evoked increases in the inspiratory motor output and hypoxia-evoked sighing.

## Introduction

The *Locus Coeruleus* (LC) is the principal noradrenergic nucleus (A6 neurons) in the CNS^1^. These neurons send extensive projections throughout the neuraxis and are implicated in the control of many homeostatic functions^2–5^, including chemosensory control of breathing^6–8^. In this regard, A6 neurons of rats respond to hypercapnic stimulation of central chemoreceptors *in vivo*^9^ and a large proportion of them are found to be intrinsically sensitive to CO_2_/[H^+^] *in vitro*^8,10–12^. In conscious rats, a permanent chemical lesion of A6 neurons significantly reduced the CO_2_-induced ventilatory response^13^.

A6 neurons also respond to activation of peripheral chemoreceptors using hypoxia^9,14^. However, a permanent chemical lesion of A6 neurons did not affect the ventilatory response to systemic hypoxia in conscious rats^15^, suggesting that such neurons play no major role in the respiratory responses evoked by activation of peripheral chemoreceptors. However, experimentally determining a causal role for A6 neurons in promoting and maintaining chemosensory control of breathing has remained elusive using traditional chemical ablation approaches, since a permanent loss of neuronal populations may be compensated for by other parts of the neuronal circuitry.

Breathing at rest is characterized by three phases: inspiration, post-inspiration and passive expiration^16^. The pre-Bötzinger Complex (pre-BötC) is responsible for generating inspiratory rhythm^16^. The neural mechanisms underlying post-inspiration are attributed to a distributed network involving lung mechanoreceptors, pontine circuits and a medullary conditional oscillator so-called “post-inspiratory complex”^17–19^. Under conditions of high chemical drive (activation of central and/or peripheral chemoreceptors), abdominal (Abd) motor activity is recruited at the end of expiration (active expiration)^20–22^. This is a mechanism to contract Abd muscles phasically to force exhalation, which assists in reducing expiratory time to promote enhanced pulmonary ventilation $({\dot{V}}_{{\rm{E}}})$^23^. Active expiration depends on a conditional active expiratory oscillator within the ventral medullary parafacial Respiratory Group (pFRG)^20–22,24^. High chemical drive also produces differing degrees of both alertness and arousal^25^, which is highly associated with A6 neuronal activation^5^. It has been proposed that the pons modulates central generation of Abd active expiration under high chemical drive^20^. A6 neurons project to the ventral medulla^26^, which we propose to modulate the activity of expiratory neurons located in the pFRG or even those determining the activity of spinal Abd motoneurons in the caudal Ventral Respiratory Group (cVRG). Given their role in mediating arousal^5^, we hypothesise that A6 neurons are a part of a vigilance centre (LC) for controlling breathing under high chemical drive and that this will include recruitment of both active inspiration and expiration in readiness for flight or fight.

The development of pharmacogenetic tools provides a neuronal phenotype specific way to acutely and reversibly inhibit the activity of selected neuronal populations^27^. Therefore, to determine the role of the LC-noradrenaline (NA) system for both resting, inspiratory and the expiratory (active expiration) responses to activation of central and peripheral chemoreceptors in conscious adult rats, we studied the effects of inhibiting A6 neurons using the insect peptide allatostatin (Alst) following their transduction with a lentiviral construct to express the G-protein-coupled Drosophila allatostatin receptor (AlstR). Activation of AlstR produces a strong sustained neuronal hyperpolarisation via the opening of inwardly rectifying K^+^ channels^28,29^, rendering them inactive.

## Results

### A6 transduction efficacy

**Figure 1:**
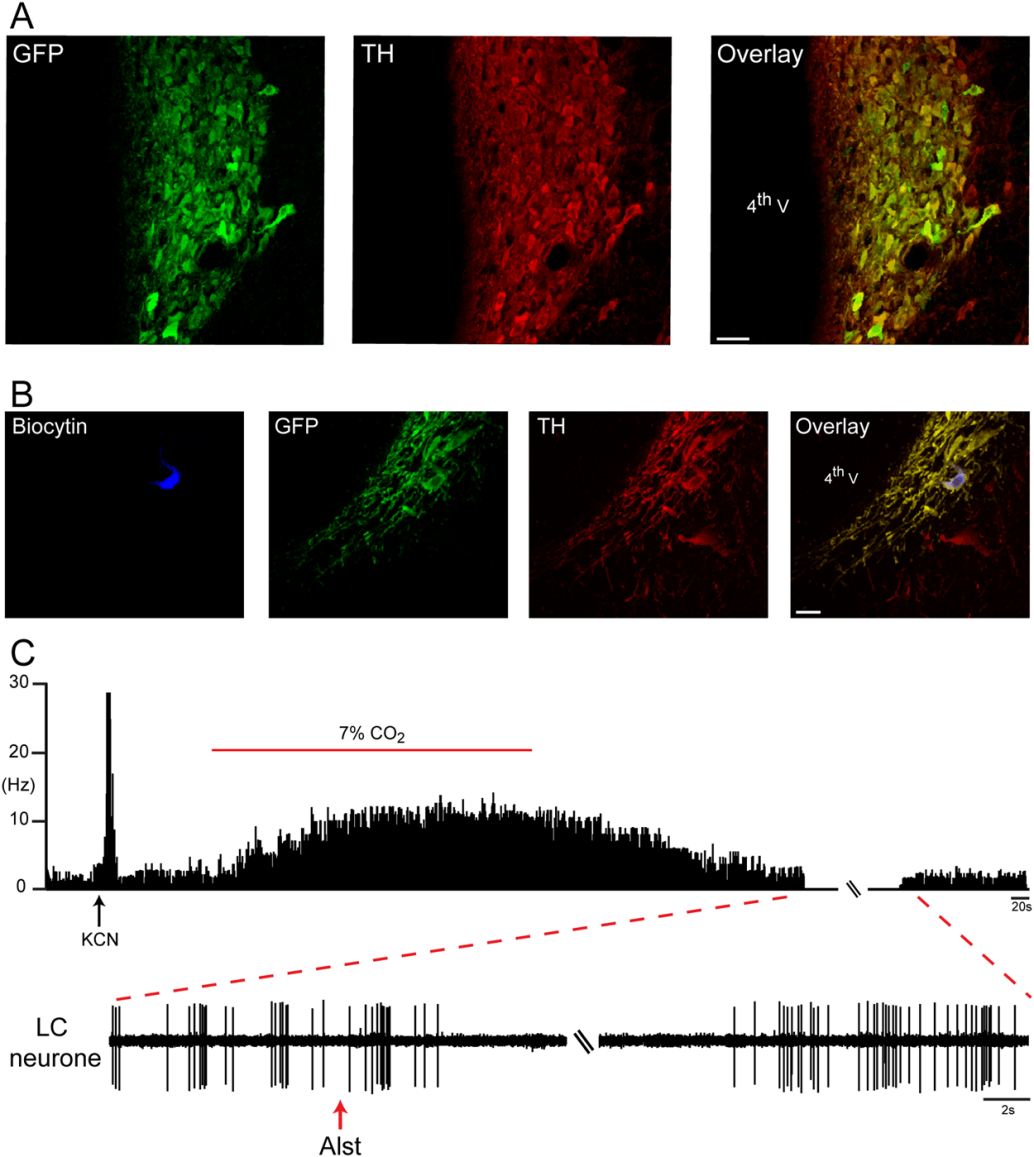
Selective and functional expression of AlstR in rat A6 neurons. Fifteen days after LC injection of PRSx8-AlstR-GFP-LVV, there is robust selective expression in noradrenergic neuronal somata and processes. GFP expression (green), TH immunoreactivity (red), and overlaid images (right). 4^th^V: fourth ventricle. Scale bar: 50 µm. (**B**) One neuron juxtacellular labelled *in vivo* with biocytin, exhibiting GFP and TH immunofluorescence (noradrenergic LC neuron). Scale bar: 20 µm. (**C**) Instantaneous firing frequency (bin width 10 ms) extracellularly recorded from the same labelled A6 neuron during baseline condition and in response to activation of peripheral chemoreceptors using KCN. This neuron also increased its firing frequency to 7% CO_2_. Intracerebroventricular application of Alst rapidly and reversibly abolished its firing frequency.

Noradrenergic brainstem neurons express the transcriptional factor Phox2 and can be targeted using lentiviral vectors (LVV) to express gene of interest under the control of an artificial promoter - PRSx8^30,31^. Direct bilateral stereotaxic injection of PRSx8-AlstR-GFP-LVV into the LC produced selective expression of the fluorophore-AlstR construct within immunocytochemically characterized LC neurons (Fig. 1A). These transduced neurons showed characteristic membrane-delimited fluorescence with strong signal from the dendrites and axonal processes, as expected of the receptor-fluorophore construct. The expression was limited to neurons that were identified as noradrenergic (A6 neurons) on the basis of their tyrosine hydroxylase (TH) immunofluorescence (Fig. 1A). Fluorophore-AlstR construct was expressed in 91 ± 1.7% of A6 neurons at the entire LC (9.16 to 10.32 mm caudal to the bregma; n = 32), demonstrating the specificity of viral targeting of the A6 neurons.

### Single A6 Unit Recording

The effect of AlstR activation on spontaneous electrical activity of A6 neurons was tested in PRSx8-AlstR-GFP-LVV anaesthetised rats (Fig. 1B,C). A6 neurons, expressing GFP, had a mean firing frequency of 3.22 ± 0.41 Hz (n = 4), which was similar to that observed in previous studies^32,33^. Acute activation of peripheral (intravenous injection of potassium cyanide - KCN; p < 0.0001) or central chemoreceptors (7% CO_2_; p = 0.003) significantly augmented their basal firing frequency (26.76 ± 2.57 and 8.8 ± 0.81 Hz, respectively). These neurons were then recorded during application of Alst into the lateral ventricle, which reversibly abolished their firing frequency within ~1 min of delivery in every cell tested (Fig. 1C).

### Respiratory responses to Alst-induced inhibition of A6 neurons

#### Spontaneous breathing

**Figure 2:**
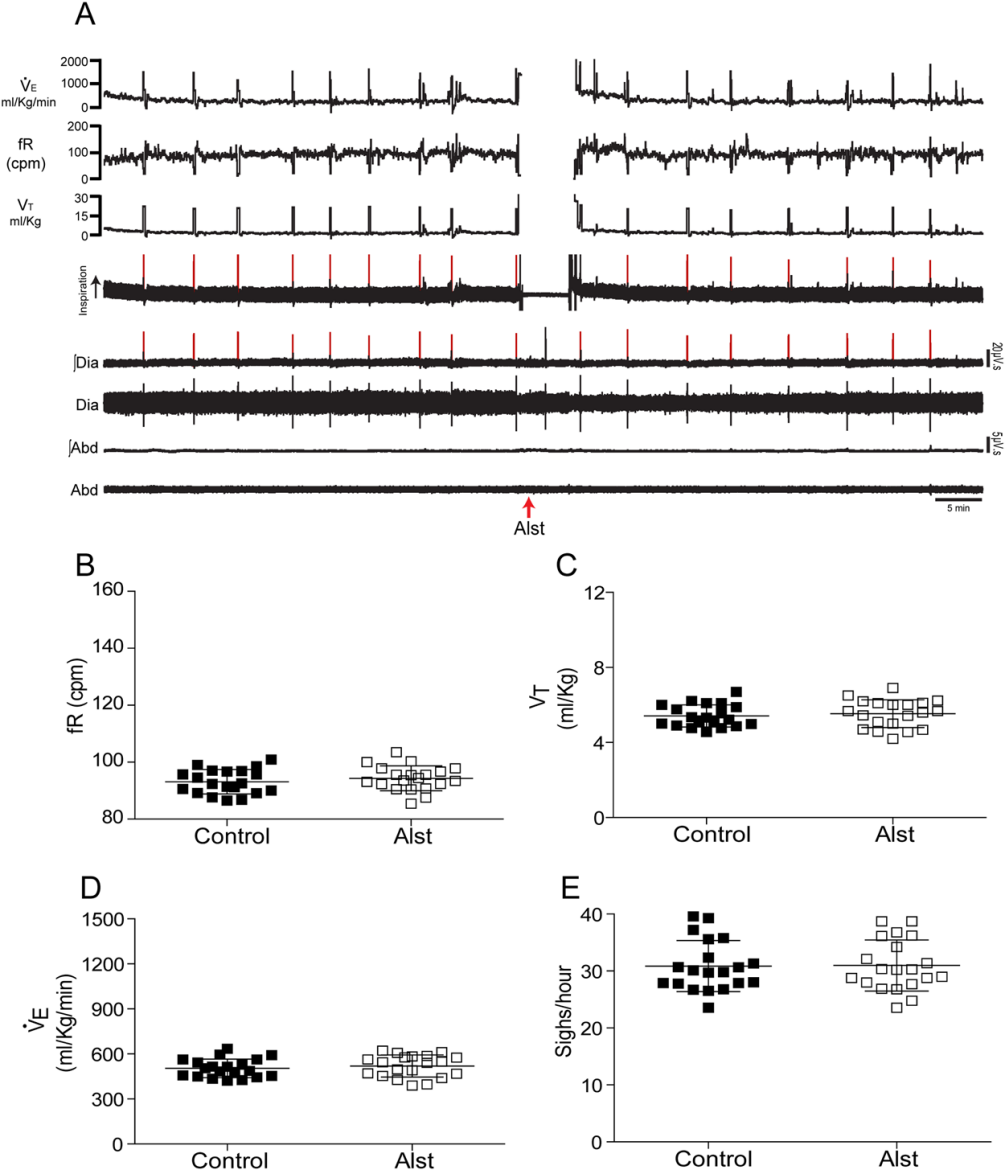
Acute inhibition of A6 neurons and effect on the baseline ventilatory, inspiratory and expiratory parameters of conscious adult rats. (**A**) Raw and integrated (∫) records of Dia_EMG_ and Abd_EMG_ activities and barometric respiratory movements, as well as fR, V_T_ and V_E _data from one rat, in which the LC was transduced with PRSx8-AlstR-GFP-LVV before and after Alst application into the lateral ventricle. Note the absence of changes in baseline inspiratory and expiratory activities, in the ventilatory parameters, as well as in the number of sighs (red lines) after acute inhibition of A6 neurons. Summary of data showing the changes in the fR (**B**), V_T_ (**C)**, VE (**D**) and in the number of sighs (**E**) after application of Alst.

We next investigated whether acute inhibition of A6 neurons would generate changes in inspiratory and expiratory activities, as well as in ventilatory parameters of conscious rats under baseline conditions (room air exposure; n = 20). Figure 2A shows representative raw and integrated records of electromyogram (EMG) from diaphragm (Dia_EMG_) and Abd_EMG_ muscles, barometric respiratory movements, as well as respiratory frequency (fR), tidal volume (V_T_) and ${\dot{V}}_{{\rm{E}}}$ data from one rat in which the LC was transduced with PRSx8-AlstR-GFP-LVV. Active expiration was never observed during baseline conditions, i.e. no rhythmic expiratory Abd_EMG_ activity was present. On the other hand, every two to three minutes we observed a sigh (a high-amplitude inspiratory breath; red lines of Fig. 2A) in Dia_EMG_ activity and in barometric respiratory movement. The grouped data show that acute inhibition of A6 neurons using Alst application into the lateral ventricle did not affect Dia_EMG_ amplitude (10.21 ± 0.72 vs 10.23 ± 0.76 µV), Abd_EMG_ expiratory activity (2.02 ± 0.35 vs 2 ± 0.38 µV), ventilatory parameters [(fR: 94.33 ± 4.37 vs 93.12 ± 4.3 cpm) (V_T_: 5.53 ± 0.74 vs 5.41 ± 0.59 ml/Kg) (${\dot{V}}_{{\rm{E}}}$: 519.6 ± 73.67 vs 504.3 ± 61.9 ml/Kg/min)] or sighing (30.97 ± 4.48 vs 30.86 ± 4.48 sighs/hour) of rats exposed to room air (Fig. 2A–E). Thus, A6 neurons are not involved in control of baseline breathing in adult conscious rats.

#### Hypoxic stimulation of peripheral chemoreceptors

**Figure 3:**
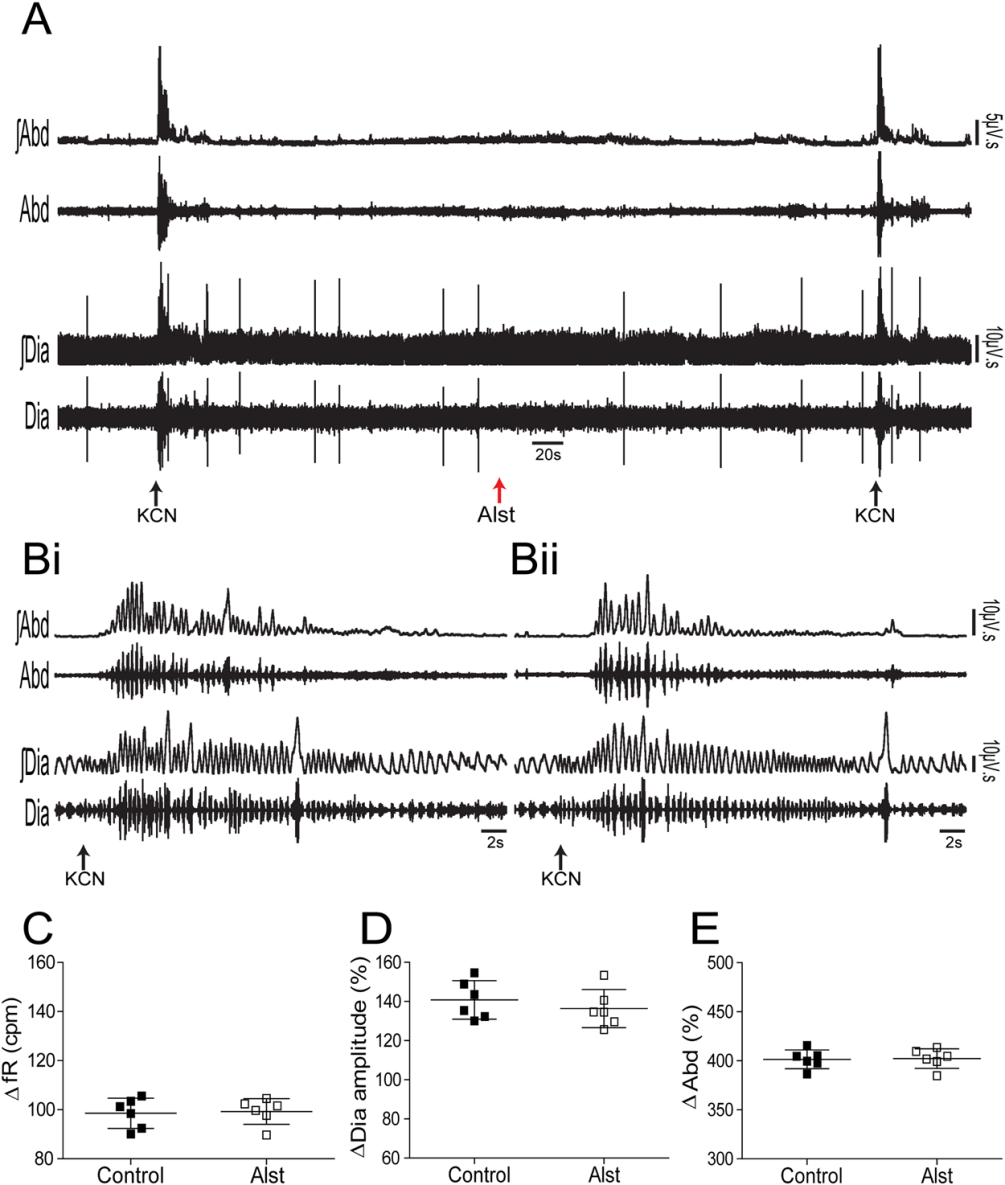
Acute inhibition of A6 neurons and effect on the inspiratory and expiratory responses to activation of peripheral chemoreceptors of conscious adult rats. (**A**) Raw and integrated (∫) records of Dia_EMG_ and Abd_EMG_ activities from one representative rat in which the LC was transduced with PRSx8-AlstR-GFP-LVV. The inspiratory and expiratory responses to peripheral chemoreceptor activation using KCN, before and after Alst application into lateral ventricle, are shown. Magnification of baseline and reflex inspiratory and expiratory responses from the same rat before (Bi) and after (Bii) Alst application. Note the absence of changes in the peripheral chemoreflex-induced inspiratory and expiratory responses after acute inhibition of A6 neurons. Summary of data showing the changes in the reflex responses of fR (**C**), Dia_EMG_ amplitude (**D**) and Abd_EMG_ (**E**) after application of Alst.

**Figure 4:**
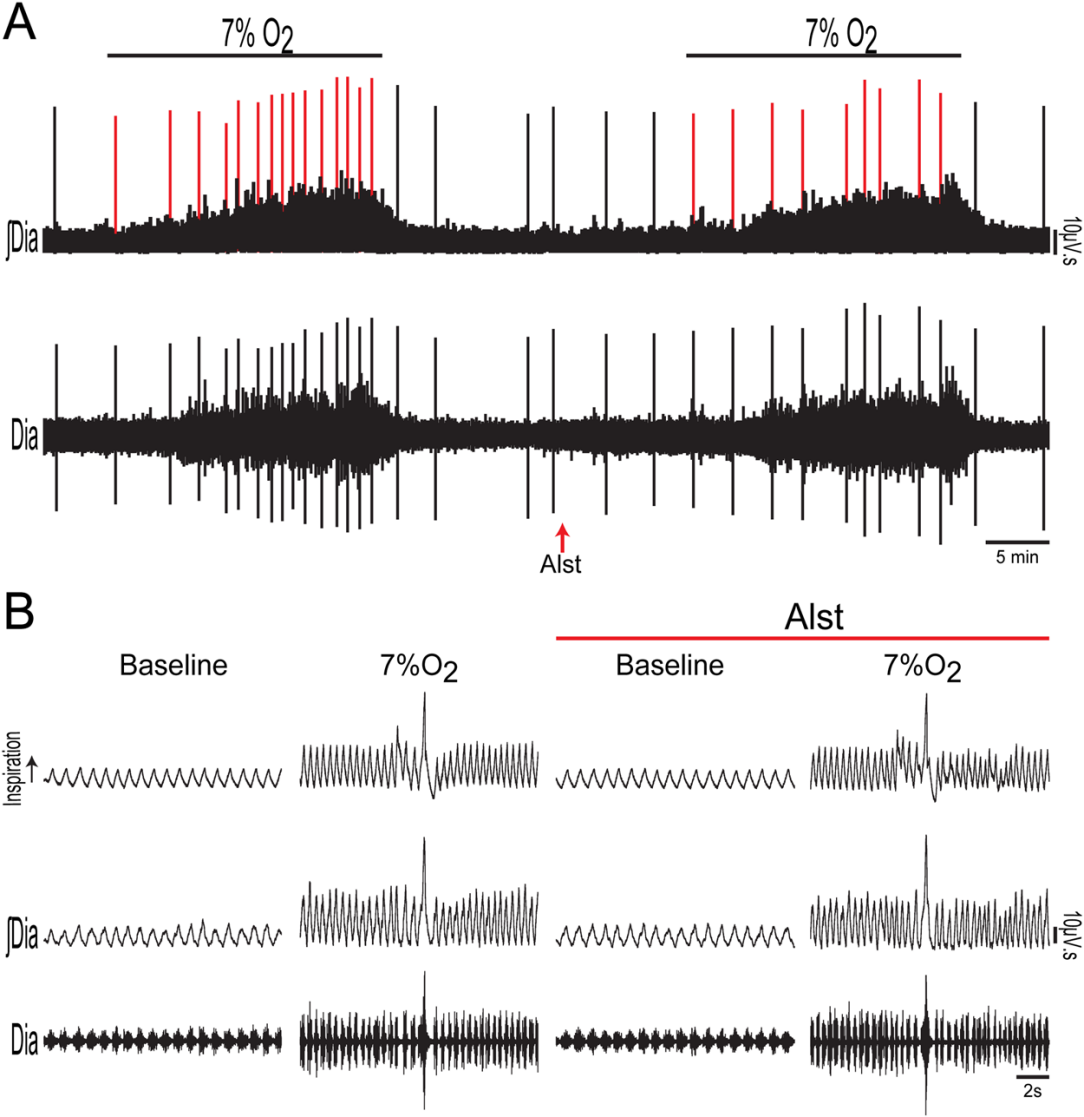
Acute inhibition of A6 neurons and effect on the inspiratory and ventilatory responses to systemic hypoxic hypoxia of conscious adult rats. (**A**) Raw and integrated (∫) records of Dia_EMG_ activity from a rat in which the LC was transduced with PRSx8-AlstR-GFP-LVV, illustrating the changes in inspiration and in the number of sighs (red lines) induced by systemic hypoxic hypoxia (7% O_2_) before and after Alst application into lateral ventricle. (**B**) Magnification of baseline and reflex inspiratory and ventilatory (barometric respiratory movements) responses from the same rat before and after Alst application. Note the absence of changes in the hypoxia-induced inspiratory and ventilatory responses, but the significant reduction in the number of sighs, after acute inhibition of A6 neurons.

**Figure 5:**
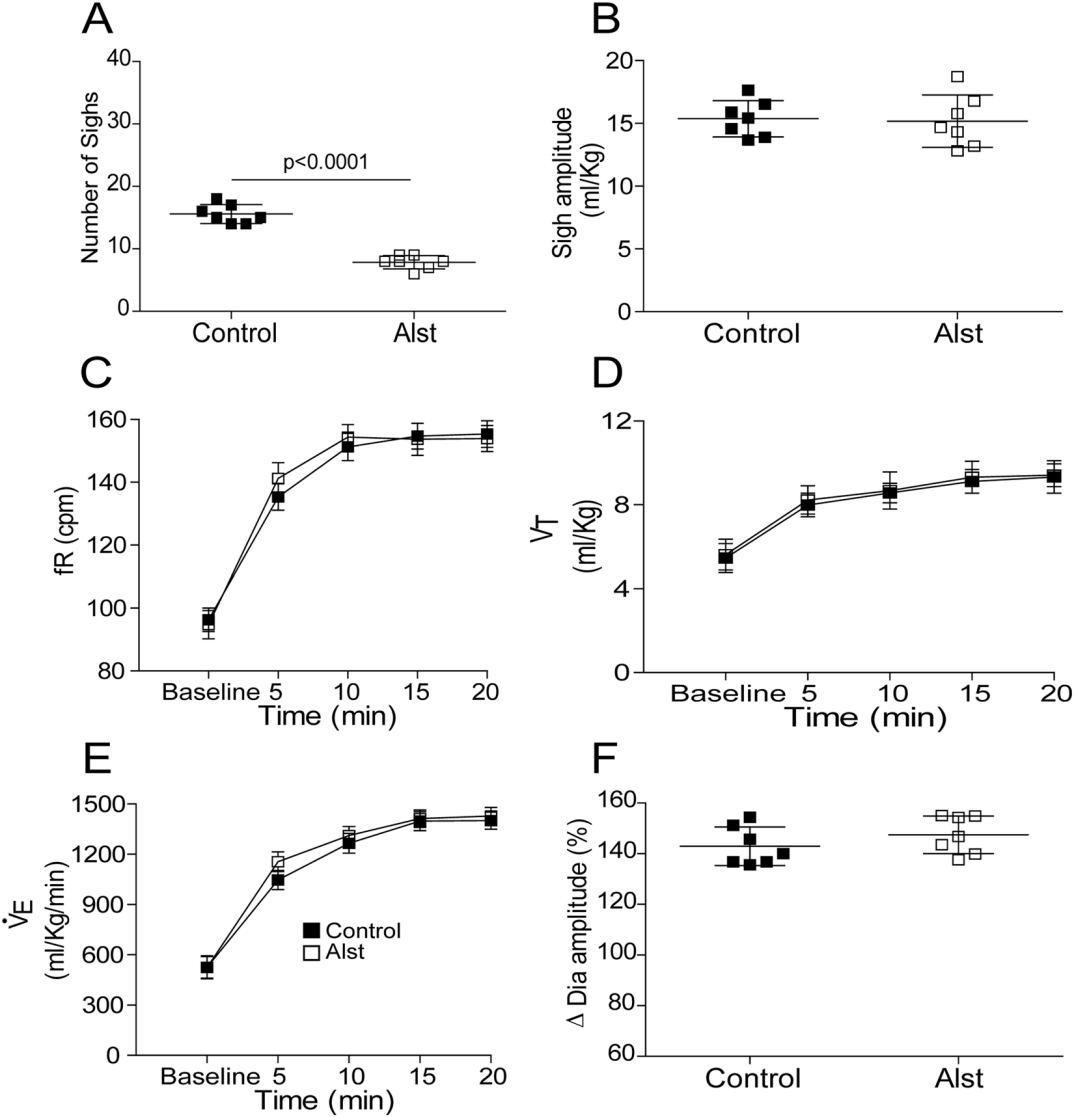
Acute inhibition of A6 neurons and effect on the respiratory responses to systemic hypoxic hypoxia of conscious adult rats. Summary of data showing the changes in the responses of the number of sighs (**A**), sigh amplitude (**B**), fR (**C**), V_T_ (**D)**, VE (**E**) and Dia_EMG_ amplitude (**F**) to systemic hypoxic hypoxia after application of Alst in rats in which the LC was transduced with PRSx8-AlstR-GFP-LVV.

In order to investigate whether A6 neurons affect breathing under conditions of high chemical drive, we first activated the peripheral chemoreceptors using intravenous injection of KCN to mimic cytotoxic hypoxia (n = 6). Figure 3A shows the peripheral chemoreflex-induced inspiratory and expiratory responses from one rat transduced with PRSx8-AlstR-GFP-LVV into LC. Acute activation of peripheral chemoreceptors increased fR and Dia_EMG_ amplitude, as well as evoked active expiration in Abd_EMG_ (Fig. 3A-Bi). Following inhibition of A6 neurons these responses remained unchanged [(inspiratory = Δ fR: 99.25 ± 5.24 vs 98.54 ± 6.16 cpm; Δ Dia_EMG_ amplitude: 136.4 ± 9.76 vs 140.8 ± 9.81%) (expiratory = Δ Abd_EMG_: 402.2 ± 10 vs 401.5 ± 9.6%); Fig. 3B–E]. However, the sighing rate (red lines of Fig. 4A) was reduced to 7.85 ± 1.06 from 15.57 ± 1.51 (p < 0.0001) during 20 min of systemic hypoxic hypoxia (7% O_2_; n = 7), but sigh amplitude was unchanged (15.18 ± 2.08 vs 15.37 ± 1.44 ml/Kg; Figs 4A and 5A,B). Systemic hypoxic hypoxia also increased fR, Dia_EMG_ amplitude, V_T_ and ${\dot{V}}_{{\rm{E}}}$, but did not evoke active expiration (data not shown). Alst application did not modify these peripheral chemoreceptor-evoked reflex responses [at 20 minutes; (fR: 153.89 ± 4.11 vs 155.34 ± 4.23 cpm) (Dia_EMG_ amplitude: 147.4 ± 7.4 vs 142.9 ± 7.59%) (V_T_: 9.41 ± 0.54 vs 9.33 ± 0.77 ml/Kg) (${\dot{V}}_{{\rm{E}}}$: 1428.49 ± 50.89 vs 1400.1 ± 51.24 ml/Kg/min); Figs 4A,B and 5C–F]. Thus, A6 neurons are involved in hypoxia-induced sighing, but not in other to systemic hypoxic hypoxia-evoked respiratory responses in conscious adult rats. Alst produced no effect on sighing rate (15.5 ± 2.08 vs 14.75 ± 3.8; n = 4) in rats transduced with PRSx8-GFP-LVV (control virus) during systemic hypoxic hypoxia.

#### Hypercapnic stimulation of central chemoreceptors

**Figure 6:**
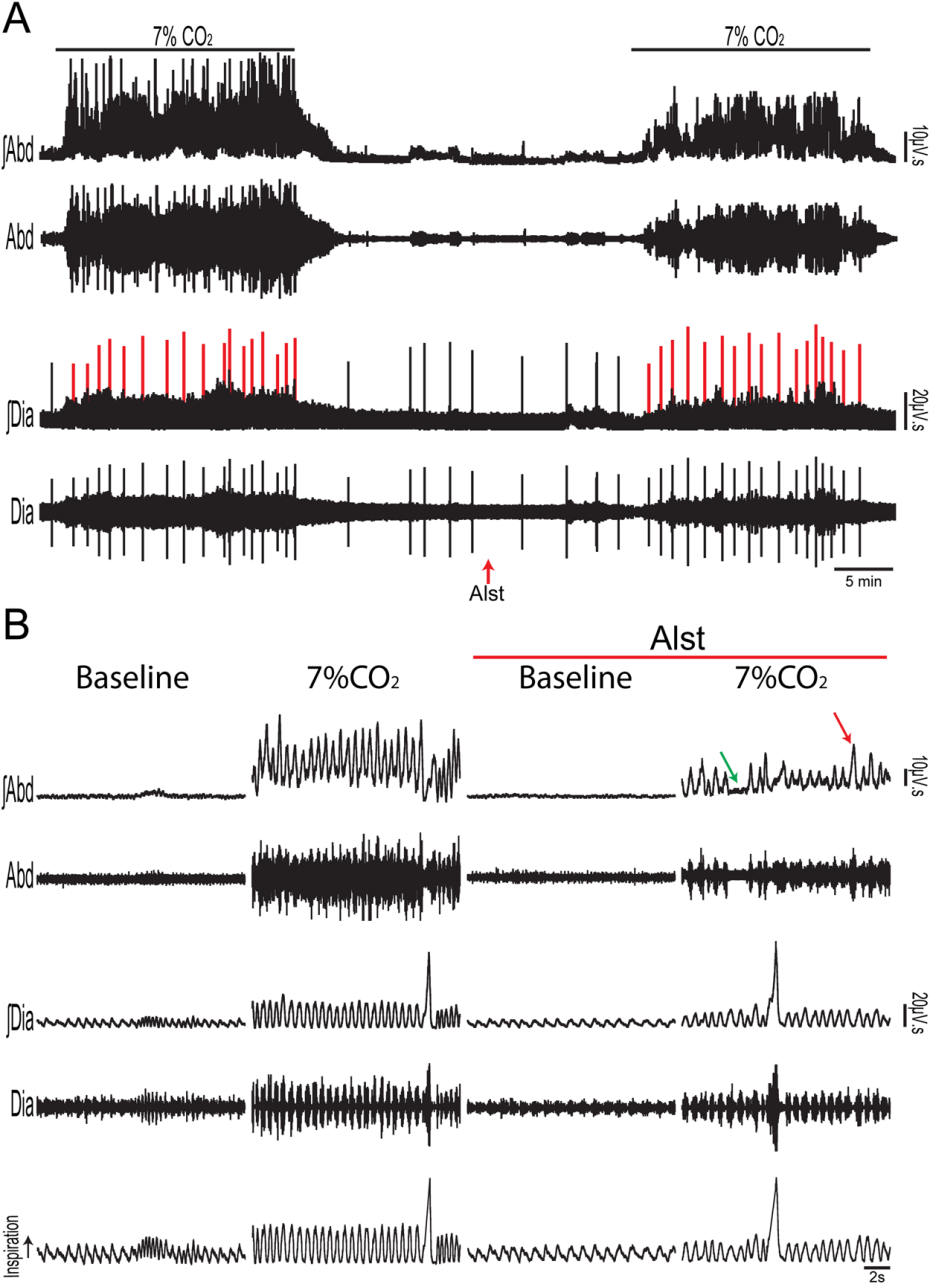
Acute inhibition of A6 neurons and effect on the inspiratory, active expiratory and ventilatory responses to stimulation of central chemoreceptors in conscious adult rats. (**A**) Raw and integrated (∫) records of Dia_EMG_ and Abd_EMG_ activities from one animal in which the LC was transduced with PRSx8-AlstR-GFP-LVV. Note the changes in inspiration, expiration and in the number of sighs (red lines) induced by activation of the central chemoreceptor (7% CO_2_), before and after Alst application into lateral ventricle. (**B**) Magnification of baseline and reflex inspiratory, expiratory and ventilatory (barometric respiratory movements) responses from the same rat before and after Alst application. Note that Alst application reduced significantly the fR, Dia_EMG_ amplitude, the Abd_EMG_ active expiration incidence (green arrow: absence of active expiration; red arrow: active expiration) and magnitude, as well as the ventilatory parameters.

**Figure 7:**
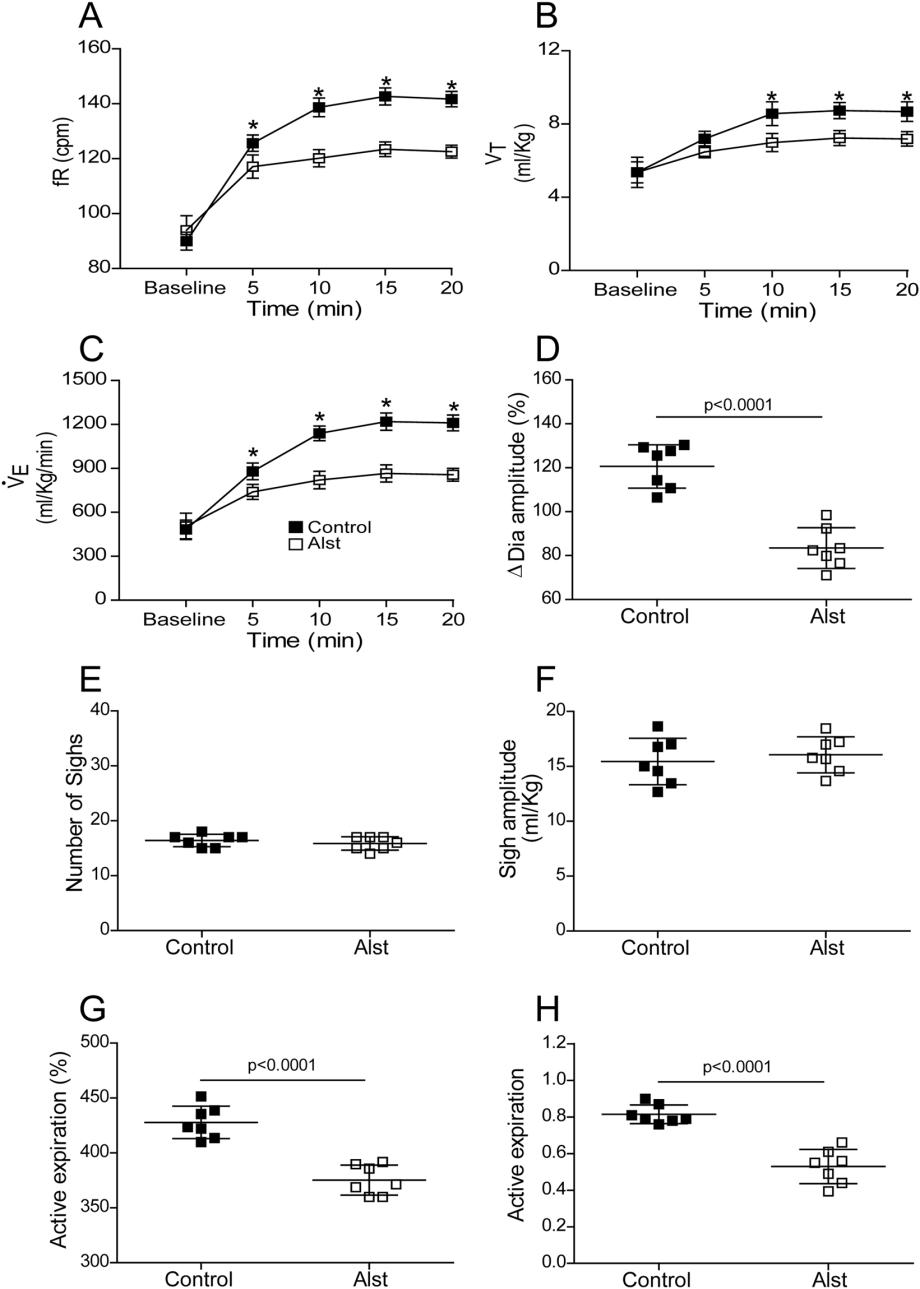
Acute inhibition of A6 neurons and effect on respiratory responses to activation of central chemoreceptors of conscious adult rats. Summary of data showing the changes in the reflex responses of the fR (**A**), V_T_ (**B)**, VE (**C**), Dia_EMG_ amplitude (**D**), number of sighs (**E**), sigh amplitude (**F**), active expiration magnitude (**G**) and active expiration incidence (**H**; number 1 means that Abd_EMG_ active expiration is at its maximal value established in each animal during hypercapnia – see Methods) after application of Alst in conscious adult rats in which the LC was transduced with PRSx8-AlstR-GFP-LVV. *p < 0.0001.

Hypercapnia (20 min of 7% CO_2_; n = 7) increased Dia_EMG_ amplitude, the number of sighs, fR (by reducing DI and DE), V_T_, ${\dot{V}}_{{\rm{E}}}$ and evoked active expiration in Abd_EMG_ of PRSx8-AlstR-GFP-LVV transduced rats, in which carotid peripheral chemoreceptors had been denervated (Figs 6A,B and 7A–H). Active expiration was prevalent as noted by the high values of expiratory Abd_EMG_ incidence (Fig. 7H) in response to activation of central chemoreceptors. Alst application reduced the central chemoreceptors-induced responses of fR (at 20 minutes: 122.56 ± 2.31 vs 141.67 ± 2.78 cpm; p < 0.0001), V_T_ (at 20 minutes: 7.18 ± 0.39 vs 8.67 ± 0.53 ml/Kg; p < 0.0001), ${\dot{V}}_{{\rm{E}}}$ (at 20 minutes: 856.79 ± 43.79 vs 1210.68 ± 53.56 ml/Kg/min; p < 0.0001) and Dia_EMG_ amplitude (at 20 minutes: 83.42 ± 9.3 vs 120.6 ± 3.73%; p < 0.0001), but did not change either the number of sighs (15.86 ± 1.21 vs 16.43 ± 1.13) or their amplitude (16.05 ± 1.64 vs 15.45 ± 2.11 ml/Kg; Figs 6A,B and 7A–F). Alst also reduced both the incidence (0.52 ± 0.09 vs 0.81 ± 0.05; p < 0.0001) and magnitude of Abd_EMG_ active expiration (375.3 ± 13.64 vs 427.8 ± 14.76%; p < 0.0001; Figs 6A,B and 7G,H). On the other hand, Alst application increased duration of expiration (DE; at 20 minutes: 0.30 ± 002 vs 0.26 ± 0.03; p = 0.01) and duration of inspiration (DI; at 20 minutes: 0.20 ± 0.01 vs 0.18 ± 0.02; p = 0.03; Fig. 6A,B). Alst produced no effect on fR (at 20 minutes: 142.5 ± 3.33 vs 146.44 ± 3.59 cpm), V_T_ (at 20 minutes: 8.89 ± 0.77 vs 8.19 ± 0.86 ml/Kg), ${\dot{V}}_{{\rm{E}}}$ (at 20 minutes: 1200.56 ± 67.55 vs 1248.44 ± 59.44 ml/Kg/min) or Abd_EMG_ active expiration magnitude (419.1 ± 15.44 vs 437.55 ± 22.44%) in rats transduced with PRSx8-GFP-LVV (n = 4) during hypercapnia.

To determine the contribution of the reduction in active expiration after acute inhibition of A6 neurons on ${\dot{V}}_{{\rm{E}}}$, we evaluated the ventilatory parameters at different times after Alst application during hypercapnia in carotid body denervated rats, i.e. in the presence (red arrow - Fig. 6B) and absence (green arrow - Fig. 6B) of Abd_EMG_ active expiration. Alst application reduced fR, V_T_ and ${\dot{V}}_{{\rm{E}}}$, but increased DI and DE, regardless of whether Abd_EMG_ active expiration was present. However, reductions in fR, V_T_ and ${\dot{V}}_{{\rm{E}}}$, and the increase in DE, were greater in the absence of active expiration than during its presence [(fR: −12.79 ± 3.21 vs −9.48 ± 1.32%; p = 0.02) (DE: 11.25 ± 2.39 vs 8.2 ± 2.11%; p = 0.02) (V_T_: −15.34 ± 3.61 vs −10.54 ± 2.34%; p = 0.01) (${\dot{V}}_{{\rm{E}}}$: −26.39 ± 4.11 vs −18.67 ± 3.99%; p = 0.003), suggesting that the reduction in ${\dot{V}}_{{\rm{E}}}$ is due to reductions in both active inspiratory and expiration responses.

## Discussion

This report reveals a novel and essential functional role of A6 neurons in the chemosensory control of inspiration and expiration in adult conscious rats. In the absence of peripheral chemoreceptors, inhibition of A6 neurons reduced the magnitude and incidence of both the CO_2_-evoked Abd_EMG_ active expiration and Dia_EMG_ inspiratory activity (frequency and amplitude). These changes in both inspiratory and expiratory activities were responsible for the observed reduction in ${\dot{V}}_{{\rm{E}}}$ during hypercapnia when A6 neuronal activity was pharmacologically inhibited. However, inhibition of A6 neurons had no effect on resting or peripheral chemoreflex-evoked respiratory activities and ${\dot{V}}_{{\rm{E}}}$, although the number of sighs evoked by systemic hypoxic hypoxia was reduced. Together, these data indicate that A6 neurons are a part of a vigilance centre for controlling breathing under high chemical drive and that this includes a CO_2_-dependent augmentation of inspiratory and active expiratory drive and hypoxia-evoked sighing.

Rapid inhibitory and reversible effects of Alst were reported previously to be selective for AlstR-transduced neuron^28,29^. In the present study, we confirmed effective silencing of AlstR-expressing A6 neurons by Alst during single unit extracellular recording in anaesthetised rats. Thus, the physiological data on perturbations of respiratory pattern reported herein, together with our cellular-level electrophysiological evidence provided, suggest that Alst application effectively inhibits a significant proportion of transduced A6 neurons *in vivo*.

The present study also demonstrated that inspiratory and expiratory activities, and ${\dot{V}}_{{\rm{E}}}$ were unaffected by Alst under eupnoeic breathing, suggesting that A6 neurons play no role in the control of breathing in conscious rats at rest while breathing room air. Similar results regarding ventilatory parameters have been demonstrated in previous studies using permanent chemical lesions of A6 neurons in rats^13,15^. Likewise, acute silencing of these neurons did not affect the respiratory responses to activation of peripheral chemoreceptors using either cytotoxic or hypoxic hypoxia. Although previous studies indicate that A6 neurons: (i) receive excitatory synaptic drive from the central respiratory network, (ii) are responsive to activation of peripheral chemoreceptors and (iii) are intrinsically sensitive to low levels of O_2_^10,14,34,35^, the present study ruled out a possible contribution of these neurons for the inspiratory and expiratory responses to activation of carotid body chemoreceptors. We propose that A6 neurons are involved in both the behavioural arousal and respiratory-modulated response of the sympathetic outflow to hypoxia, since these neurons are excited, along with sympathetic outflow, during peripheral chemoreceptor stimulation, and exhibit a pronounced respiratory pattern under conditions of strong entrainment of the sympathetic vasomotor outflow by the central respiratory network^14^.

**Figure 8:**
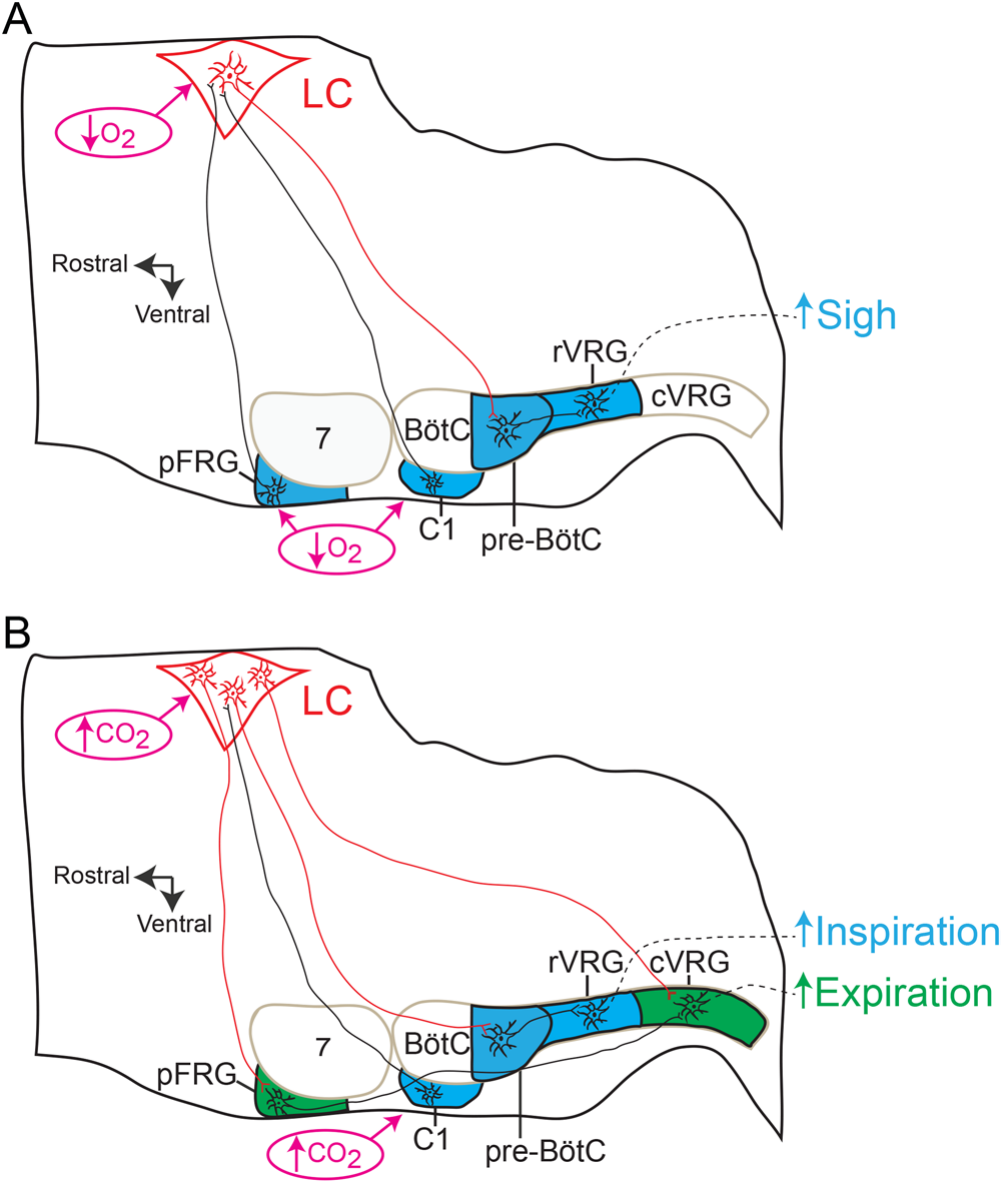
Schematic depicting proposed neural mechanisms by which A6 neurons regulate inspiratory and expiratory responses to high chemical drive. Parasagittal views of the brainstem showing the location of the medullary ventral respiratory group and C1 catecholaminergic region, as well as the pontine LC A6 neurons (red) and Facial Motor Nucleus (VII). The respiratory rhythmogenic sites of the pre-Bötzinger Complex (pre-BötC, inspiratory neurons) and parafacial Respiratory Group (pFRG, for active expiration and sigh), as well as the expiratory Bötzinger Complex (BötC), rostral Ventral Respiratory Group (rVRG; containing inspiratory bulbo-spinal premotor neurons) and caudal Ventral Respiratory Group (cVRG; containing expiratory bulbo-spinal premotor neurons) are also shown. (**A**) Sigh response during systemic hypoxia: A6 neurons might be directly activated by systemic hypoxia (↓ O_2_)^34,35^ or by sigh-promoting bombesin-like pFRG^38^ and C1 catecholaminergic neurons^40^ to increase the frequency of sighing through adrenergic receptor activation of pre-BötC^42^. (**B**) Active inspiratory and expiratory responses during hypercapnia: A6 neurons, activated by CO_2_^7^ or C1 catecholaminergic neurons^59^, enhance inspiration and V_E_ through adrenergic receptor activation of pre-BötC^52,53^. A6 neurons might also provide either tonic^9^ or expiratory-related^14^ excitatory input to the conditional expiratory oscillator located in the pFRG or directly to the cVRG, for onward relay to expiratory spinal motoneurons, enhancing active expiration.

Sighing during normal breathing has been hypothesised to prevent lung atelectasis, maintaining lung compliance and improving alveolar oxygenation^36^. Sighing increases in response to hypoxia and hypercapnia^37,38^. The data of the present study demonstrate the crucial role of A6 neurons for driving sighs induced by systemic hypoxic hypoxia, but not during hypercapnia or eupnoeic breathing. Recent studies have shown that sighing is controlled by two bombesin-like neuropeptide pathways, neuromedin B and gastrin-releasing peptide, expressed in the pFRG neurons, which mediate signalling with the pre-BötC^38^. A6 neurons receive a dense network of immunoreactive fibres expressing bombesin-like neuropeptides^39^. Therefore, it is reasonable to suggest that A6 neurons might be activated by bombesin-like neuropeptides from pFRG during hypoxia to increase the frequency of sighing (Fig. 8A). A6 neurons may also be activated by other sigh-promoting regions responsive to hypoxia, such as the ventral medullary C1 catecholaminergic neurons^40^ (Fig. 8A). This is a group of neurons with dual catecholaminergic/glutamatergic phenotype^41^ that modulate the activity of inspiratory pre-BötC neurons during hypoxia by local release of NA. Adrenergic receptor activation of the pre-BötC induces sighs by increasing the frequency of intrinsically bursting pacemaker neurons that rely on a persistent sodium current^42^. The sigh-specific effect of A6 neurons is unique, since no A6 mediated effects were observed on eupnoeic inspiratory activity and ${\dot{V}}_{{\rm{E}}}$ or for the inspiratory responses to either cytotoxic or systemic hypoxic hypoxia. This is clinically relevant since sighs play a critical role in the sequence of events that lead to arousal^25,43^. The majority of arousals during high chemical drive, or even during natural sleep, occur as a stereotypic sequence that begins with a sigh, followed by a startle and subsequent cortical arousal^44–46^; the latter being a well-known function of A6 neurons^5^. Thus, a failure to generate sighs during the hypoxic condition may contribute to the events that lead to Sudden Infant Death Syndrome (SIDS)^47,48^. This hypothesis is supported by finding that sighs and arousals are disturbed in SIDS patients^49^. On the other hand, inhibition of A6 neurons did not affect sighs induced by hypercapnia, suggesting the existence of at least two separate neural circuits controlling sighing. Whether the activation of other catecholaminergic neurons (e.g. C1 or A5 neurons) mediates the hypercapnic sigh response remains an open question.

It is well established that LC participates in central chemoreception to CO_2_/[H^+^]^7^. Hypercapnia/acidosis increases the firing frequency of these neurons both *in vivo* as well as in *in* v*itro* preparations of adult rats^8,9^. Previous studies have employed subtractive methods to study the role of the A6 neurons in CO_2_ regulation of breathing. These methods using toxins to inhibit the noradrenergic system showed different magnitudes of inhibition of central chemoreception^13,50,51^. This may, in part, be ascribed to the long-term adaptation to the loss of a population of neurons. Our findings clearly show a crucial involvement of A6 neurons in the control of inspiration (fR and Dia_EMG_ amplitude) and in the generation of Abd_EMG_ active expiration (its magnitude and incidence) during stimulation of central chemoreceptors. A6 neurons project to the pre-BötC and regulate breathing^52,53^. It is possible that hypercapnia evokes A6 neurons to activate α_1_-adrenoceptors in pre-BötC^54^, thereby enhancing inspiration and ${\dot{V}}_{{\rm{E}}}$ (Fig. 8B). Several observations also suggest that this metabolic challenge could activate the A6 via C1 neurons (Fig. 8B). The C1 neurons densely innervate the LC^55,56^, C1 and A6 neurons respond to CO_2_^57^ and optogenetic stimulation of the C1 activates A6 neurons^58,59^. Hypercapnia also produce differing degrees of alertness and arousal^25^ and increased alertness is highly associated with A6 and C1 neuronal activation^5,40^. Therefore, excitatory activation transmitted from C1 to A6 neurons under hypercapnia is not hard to rationalize and this neural pathway could facilitate arousal and therefore contribute to the airway defensive responses to hypercapnia^25,45^; an issue that requires further study.

Our study unearths for the first time that A6 neurons play a major role in central generation of Abd_EMG_ active expiration to enhance ${\dot{V}}_{{\rm{E}}}$ during hypercapnia. We observed that reductions in active expiration incidence led to decreases in ${\dot{V}}_{{\rm{E}}}$. These data agree with the proposal that active expiration enhances ${\dot{V}}_{{\rm{E}}}$ by increases in both V_T_ (recruiting expiratory reserve volume) and fR, but decreases in DE and total time of the respiratory cycle^23^. Active expiration may be state-dependent being more predominant in sleep than in wakefulness during hypercapnia^60^. Although their inhibition does not affect the duration of sleep, A6 neurons are necessary for long-term wakefulness and arousal^5^. Therefore, we propose that A6 neurons’ role in generation of active expiration is in part important for maintaining vigilance whether this is to expiratory hypercapnic responses or a threatening environment. Some evidence suggest that the posture and proprioceptive inputs from the abdominal wall may also affect active expiration^61,62^. Considering that the LC presents specialized subgroups of noradrenergic neurons, projecting to prefrontal cortex, that are involved in the anxiogenic behaviours^32,33^ and that inhalation of CO_2_-enriched air can produce anxiety and fear-like behaviours^63^, we cannot rule out that changes in the behavioural responses to hypercapnia, after acute A6 inhibition, may contribute to the reductions in both active expiration magnitude and incidence through postural changes and even by Abd proprioceptive components.

Chemosensory neurons sensitive to CO_2_/[H^+^] and identified in the LC *in vivo* are either tonically active over a wide range of arterial CO_2_ levels^9^ or show different patterns of respiratory modulation in their discharge, including expiratory modulation^14^. Thus, A6 expiratory-modulated neurons may mediate the CO_2_-evoked activity recorded from Abd_EMG_. Alternatively, when arterial CO_2_ increases, A6 neurons might provide tonic excitatory input to the conditional expiratory oscillator located in the pFRG or directly to the cVRG of the medulla for onward relay to expiratory spinal Abd motoneurons (Fig. 8B); this remains to be determined. There is a dense catecholaminergic innervation in the pFRG partially overlapping, adjacent and more medial chemosensitive region (Retrotrapezoid Nucleus- RTN)^64^. Despite the recent data showing that NA in the RTN does not contribute to the respiratory response evoked by activation of central chemoreceptors in anaesthetised rats^65^, additional experiments are needed to evaluate the role of adrenergic receptors in the pFRG for hypercapnia-evoked active expiration in conscious rats. NA may affect membrane potential subthreshold oscillations of the presumable intrinsic bursting pFRG expiratory neurons^66^ during hypercapnia, thereby regulating both the incidence and magnitude of Abd_EMG_ activity. Therefore, new electrophysiological experiments are needed to evaluate the synaptic mechanisms and the role of neuromodulators^67,68^ determining the excitability of pFRG expiratory neurons during hypercapnia.

In conclusion, the present study in conscious adult rats reveals a powerful modulatory role of A6 neurons in the CO_2_-evoked increases in active expiration, the inspiratory motor output and ${\dot{V}}_{{\rm{E}}}$, as well as hypoxia-evoked sighing. The present study prompts interesting questions as to whether A6 neurons have anatomical and cellular specialisation acting on different regions of the CNS for the control of sighing, inspiration and active expiration that are independently selected and regulated by hypoxia and hypercapnia, and whether these specialized subgroups of noradrenergic neurons share their involvement with other behavioural functions (e.g. arousal and anxiety).

## Methods

### Animals

The experiments were performed on male Wistar rats provided by the Animal Care Facility at the Ribeirão Preto campus of the University of São Paulo, Brazil. All experimental protocols were approved by the Institutional Ethics Committee for Animal Experimentation at the School of Medicine of Ribeirão Preto, University of São Paulo (protocols #093/2009 and #122/2016). All methods were carried out in accordance with The Principles of Laboratory Animal Care (NIH publication no. 85Y23, revised 1996). Animals were housed with a 12 h light/dark cycle at a constant temperature (22 ± 1 °C) with *ad libitum* access to standard rat chow and water.

### Viral vectors

The LVV system used here was HIV-1-derived and pseudo-typed with the VSV-G envelope^29^. The plasmids pTYF-PRSx8-AlstR-IRES2-GFP and pTYF-PRSx8-IRES2-GFP were cloned into the LVV. Titres of PRSx8-AlstR-GFP-LVV and the control virus (PRSx8-GFP-LVV) were between 1 × 10^9^ and 1 × 10^10^ pfu. Viral concentration and titration were performed as described in detail previously^69^.

### *In vivo* gene transfer

Male rats (225–250 g) were anaesthetised with a ketamine (75 mg kg^−1^ i.p.)/xylazine (5 mg kg^−1^ i.p.) mixture. The depth of anaesthesia was checked at regular intervals (15–20 min) by assessing the withdrawal reflex response to noxious pinching of the tail or hind paw. Animals were placed in a stereotaxic frame (David Kopf, Tujunga, USA) and four microinjections (each 250 μm apart in the dorsoventral axis) per side of PRSx8-AlstR-GFP-LVV (250 nl each, over 2 min) were delivered into LC bilaterally (Picospritzer II; Parker Instruments, Cleveland, USA). The microinjections were made 2 mm caudal to bregma, ±1.2 mm lateral from the midline and 5.5–6.0 mm below the brain surface with a 10° rostral angulation. A stainless-steel guide cannula (13 mm long, 0.6 mm o.d., 0.4 mm i.d.) was implanted into the lateral cerebral ventricle (−0.6 mm to Bregma, 1.5 mm lateral to the midline and −3.6 mm ventral to dura mater). The guide cannula was fixed to the cranium using dental acrylic resin. Post-surgery, rats were treated with one prophylactic dose of analgesic and antipyretic flunixinmeglumine (1 mg/kg; Schering-Plough, Rio de Janeiro, Brazil) and 0.1 ml of veterinary antibiotic (1.2 million i.u.; Fort Dodge, Campinas, Brazil) via intramuscular injections.

### *In vivo* experiments

#### Anaesthetised studies

Experiments in anaesthetised PRSx8-AlstR-GFP-LVV rats were performed to evaluate the single cellular response of A6 neurons to activation of AlstR fifteen days after the LVV injections. General anaesthesia was induced with 5% halothane (AstraZeneca do Brasil Ltda, Cotia, Brazil) in 100% O_2_. A tracheostomy was performed and the halothane concentration was reduced to a level of 1.4–1.5%, which was sufficient to abolish the corneal reflex and the retraction of distal phalanges to strong nociceptive stimulation of the hindpaw until the end of surgery. A polyethelene catheter (PE-10 connected to PE-50; Clay Adams, Parsippany, USA) was inserted into the femoral vein for systemic administration of fluids and drug. Rats were placed onto a stereotaxic apparatus (David Kopf) on a heating pad (ALB 200 RA; Bonther, Ribeirão Preto, Brazil) and core body temperature was monitored and maintained at a minimum of 37 °C via a thermocouple (MLT1403; Harvard Apparatus, Holliston, USA). All rats were ventilated (Small animal ventilator 683; Harvard Apparatus) with 100% O_2_ throughout the experiment. End tidal-CO_2_ was monitored throughout the experiment with a capnometer (Columbus Instruments, Ohio, USA). The discharges of neurons in the LC were recorded extracellularly (Duo 773 Electrometer; World Precision Instruments, Sarasota, USA) using glass electrodes (30–50 MΩ) filled with 1.5% biocytin (Molecular Probes, Grand Island, USA) in 0.5 M sodium acetate. Effects produced by: (i) activation of peripheral chemoreceptors using an intravenous injection of KCN (50 μl; 40 μg/Kg; Merck, Darmstadt, Germany), (ii) activation of central chemoreceptors stepping end-expiratory CO_2_ from 5% to 7% and (iii) activation of AlstR on the discharge of LC neurons were evaluated. Alst (2 mM, 10 µl; Phoenix Pharmaceuticals, Inc., Burlingame, USA) was administered intracerebroventricullarly [(25 µl syringe; Hamilton Company, Reno, USA) (needle 33-gauge; Small Parts, Miami Lakes, USA)]. The correct placement of the guide cannula was confirmed at the end of the experiment by injection of Evans Blue (2% in 10 μl; Sigma-Aldrich, St. Louis, USA) and its visible presence in the intracerebroventricular system. After electrophysiological experiments, LC neurons were filled with biocytin using the previously described juxtacellular labelling method (200 ms pulses of 1.0–4.0 nA at 2.5 Hz for 1–3 min)^70^. All signals were acquired by a data acquisition system (5 KHz; CED 1401; Cambridge Electronic Design, UK) controlled by a computer running Spike 2 software (Cambridge Electronic Design).

#### Conscious studies

EMG of respiratory muscles was used to evaluate inspiratory and expiratory indexes in conscious rats^71^. Ten days after the microinjections, PRSx8-AlstR-GFP-LVV rats were re-anaesthetised (ketamine/xylazine mixture; i.p.). The depth of anaesthesia was also checked at regular intervals by assessing the withdrawal reflex response to noxious pinching of the tail or hind paw. Bipolar teflon-coated stainless steel EMG electrodes were implanted in the Dia and Abd oblique muscles, a temperature datalogger (SubCue, Calgary, Canada) was implanted within the abdominal cavity for body temperature (Tb) measurements and a polyethelene catheter was inserted into the femoral vein for systemic administration of drug. Wires were attached to an electrical socket, tunnelled under the skin and positioned on the back of the rat’s neck together with the distal end of the catheter. In the experiments involving activation of central chemoreceptors, the carotid artery bifurcations were exposed, carotid sinus nerves and all its branches sectioned^72^. The completeness of carotid body denervation was assessed on the day of the study by the absence of Dia_EMG_ and Abd_EMG_ responses to peripheral chemoreceptor stimulation using an intravenous injection of KCN. Post-surgery, rats were treated with analgesic, antipyretic and veterinary antibiotic as above. Five days later, the electrical socket of the EMG was connected to an amplifier (1700 amplifier, A-M Systems, Sequim, USA) and animals placed in a cylindrical plethysmograph chamber (5 liters). The chamber was flushed with room air at a flow rate of 1000 ml/min during baseline condition. The gas was then switched to a hypercapnic (7% CO_2_, 21% O_2_, N_2_ balance) or hypoxic (7% O_2_, N_2_ balance) mixtures for 20 minutes. Gases were mixed with a gas mixer (Pegas 4000, Columbus Instruments, Columbus, USA) using an equivalent flow rate. ${\dot{V}}_{{\rm{E}}}$, a product of V_T_ and fR, was obtained by using whole body plethysmography^73^. A volume calibration was performed by injecting a known volume of air (1 ml) inside the chamber. V_T_ was calculated using the formula described by Bartlett & Tenney^74^ and Tb was recorded using the temperature datalogger programmed to acquire every 5 min. Alst was also administered intracerebroventricullarly. The EMG (0.3–5 KHz of bandpass) and breathing-related pressure oscillations (MLT141 spirometer, ADInstruments, Bella Vista, Australia) were acquired by a data acquisition system (5 KHz; PowerLab, ADInstruments) controlled by a computer running LabChart software (ADInstruments).

### Data analysis

EMGs were recorded in absolute units (μV) and analyses were performed off-line from rectified and integrated (∫) signals (time constant: 50 ms). Dia_EMG_ burst activity was assessed as fR. DI and DE were calculated from the ${\dot{V}}_{{\rm{E}}}$ trace. A sigh was defined as a high-amplitude inspiratory breath in the barometric respiratory movements and in the Dia_EMG_ activity of at least 100% larger in amplitude than the mean amplitude of five breaths preceding each sigh. Sigh frequency was expressed as the number of sighs per hour or per protocol (systemic hypoxia or hypercapnia - 20 minutes) and sigh amplitude was measured as changes in V_T_. Changes in the Dia_EMG_ (amplitude) and Abd_EMG_ activities during baseline conditions (room air exposure) after Alst application were expressed in µV. Based upon absolute values of Dia_EMG_ and Abd_EMG_ (µV), we determined percentage changes in order to compare their activities in each animal during different experimental conditions (systemic hypoxic hypoxia and hypercapnia), before and after Alst applications. Changes in the fR and Dia_EMG_ amplitude (percentage values) in response to acute activation of peripheral chemoreceptors using KCN were assessed by the difference between baseline and the peak of response observed after the stimulus (Δ fR and Δ Dia_EMG_ amplitude). The Abd_EMG_ expiratory responses to acute peripheral chemoreflex activation was assessed by the measurement of the area under the curve, in a time window of ≤20 s after the stimulus, and expressed as percentage values (Δ Abd_EMG_ in percentage) in relation to EMG activity before the stimulus. Active expiration was defined by the presence of Abd_EMG_ expiratory activity, i.e. rhythmic burst of activity (between inspiratory Dia_EMG_ activities) above tonic levels. Event detection and the measurement of the amplitude of Abd_EMG_ active expiration was performed on LabChart software. This allowed an assessment of the incidence of active expiration. All data were normalized to their largest integrated (∫) peak amplitude of Abd_EMG_ expiratory activity obtained. The normalized values were used for comparisons of active expiration incidence during the activation of central chemoreceptors, before and after Alst application across animals (i.e number 1 being Abd_EMG_ active expiration at its maximal value observed in each animal).

### Statistical analyses

Results are expressed as mean ± SD. Data were compared using Student’s paired t test or Two-way ANOVA with Bonferroni post hoc test (GraphPad Prism 4, La Jolla, USA) in accordance with the experimental protocol. Differences were considered significant at p < 0.05.

### Histology and immunocytochemistry

Rats were killed with an overdose of anaesthetics (ketamine/xylazine mixture; i.p.) and perfused transcardially first with phosphate buffered saline (PBS; 0.1 M) and then with 4% paraformaldehyde (PFA) and brains removed and post fixed in PFA for 2–5 days. Transverse sections (40 μm thick) were cut through the LC with a cryostat (Leica CM1800; Buffalo Grove, USA) and collected into a cryoprotectant solution and stored at −20 °C before further processing. The immunofluorescence was performed with free-floating sections. Sections were blocked and permeabilized in PBS containing 10% normal horse serum and 0.5% Triton X-100 for 1 h at room temperature. After three PBS washes, the sections were incubated in primary antibodies against TH (mouse - 1:1000; Millipore, Billerica, USA) and GFP (chicken - 1:1000; AVES Labs, Tigard, USA) for 24 hrs at 4 °C. After three PBS washes, the sections were incubated in secondary antibodies Alexa 405-conjugated streptavidin (1:1000; Molecular Probes), Alexa 647-conjugated donkey anti-mouse (1:500; Molecular Probes) and Alexa 488-conjugated donkey anti-chicken (1:500; Molecular Probes) for 1 h at room temperature. Sections were washed in PBS and mounted in Fluoromount (Sigma-Aldrich). Images were collected on a Leica TCS SP5 confocal microscope equipped with 405, 488 and 633 nm laser lines and tunable emission wavelength detection. For each preparation, biocytin- and GFP-expressing neurons were identified and confocal z stacks collected sequentially for the other channel to detect the TH-expressing neurons and verify colocalization of fluorophores. GFP- and TH-expressing neurons were counted over the entire length of the LC from each rat (−9.16 to −10.32 mm from bregma). Analyses were performed using a computerized image analysis system (Image J) developed at the National Institutes of Health (https://imagej.nih.gov/ij/).
